# Complete Genome Sequence and Methylome Analysis of *Aeromonas hydrophila* Strain YL17, Isolated from a Compost Pile

**DOI:** 10.1128/genomeA.00060-16

**Published:** 2016-03-03

**Authors:** Yan-Lue Lim, Richard J. Roberts, Robson Ee, Wai-Fong Yin, Kok-Gan Chan

**Affiliations:** a Division of Genetics and Molecular Biology, Institute of Biological Sciences, Faculty of Science, University of Malaya, Kuala Lumpur, Malaysia; b New England Biolabs, Ipswich, Massachusetts, USA

## Abstract

In this report, we announce the complete genome sequence of *Aeromonas hydrophila* strain YL17. Single-molecule real-time (SMRT) DNA sequencing was used to generate the complete genome sequence and the genome-wide DNA methylation profile of this environmental isolate. A total of five unique DNA methyltransferase recognition motifs were reported here.

## GENOME ANNOUNCEMENT

Studies of prokaryotic DNA methylation are gaining major attention because of its significant implication in bacterial physiology and virulence. However, the methylomes of a vast number of bacterial organisms still remain unexplored, including *Aeromonas hydrophila*. Due to their ubiquity in the aquatic environment and their enterotoxigenic properties, *A. hydrophila* strains are extensively studied as they are increasingly regarded as emerging human pathogens (1, 2). In this study, we report the complete genome sequence of *Aeromonas hydrophila* strain YL17, a compost pile isolate, and its whole-genome and methylome analyses.

Genomic DNA extraction was performed using a MasterPure DNA purification kit (Epicentre, USA) followed by gel electrophoresis and NanoDrop spectrophotometer absorbance measurement (Thermo Scientific, USA) to assess the integrity and purity of the extracted genomic DNA. A Qubit fluorometer was used in combination with a Qubit dsDNA broad range (BR) assay kit (Invitrogen, USA) for DNA quantitation. The genomic DNA was sheared to an average size of 10-Kb using G-tubes (Covaris, USA) and was subjected to purification and concentration using Ampure PB beads (Pacific Biosciences, USA). Briefly, 5 µg of sheared genomic DNA was then proceeded with SMRTbell library construction following the “Procedure & Checklist-10-kb Template Preparation and Sequencing” protocol (http://www.pacb.com/wp-content/uploads/2015/09/Procedure-Checklist-10-kb-Template-Preparation-and-Sequencing.pdf). A Pacific Biosciences RSII sequencer (Pacific Biosciences, USA) was used to sequence the 10-Kb library by using C2 chemistry and four single-molecule real-time (SMRT) cells. 173.05-fold coverage was achieved and the reads were assembled using the Hierarchical genome assembly process (HGAP) version 2 (PacBio DevNet; Pacific Biosciences) (3). The genome sequences were successfully assembled to closure by using a seed read length of 8,000 bp and yielded a single contig (4,808,605 bp). The assembled genome was then circularized prior to annotation using the NCBI Prokaryotic Genomes Annotation Pipeline (PGAP) and Rapid Annotations using Subsystems Technology (RAST) (4).

The circularized chromosome of *A. hydrophila* strain YL12 was imported into the SMRT portal and base modification and methyltransferase motif detection and analysis were performed using the RS_Modification_and_Motif Analysis workflow (5). Six DNA methyltransferase specificities were detected as listed in Table 1. Interestingly, five motifs detected in this genome (besides G^m6^ATC) are novel and have not been reported previously. Restriction modification (RM) system annotation was carried out using the SEQWARE computer resource (6) and enabled the reliable assignment of candidate methyltransferase genes to each specificity based on their RM types and their similarity to previously characterized methyltransferase homologs (Table 1). The methylome data have been deposited in REBASE (7).

**TABLE 1:** Summary of methylation motif identified in *Aeromonas hydrophila* strain YL17 and the most likely genes responsible for each motif

Motif^a^	Percentage	Unique	Probable gene	Methylation type	RM type
G^m6^**A**TC	99.97%	No	M.AhyYL17Dam	m6A	II
CC^m6^**A**GNNNNNN**T**GAY	100%	Yes	S.AhyYL17ORF17275P	m6A	I
CC^m6^**A**YNNNNNN**T**RTC	100%	Yes	S.AhyYL17ORF670P	m6A	I
CT^m6^**A**NNNNNNNNG**T**TC	100%	Yes	S.AhyYL17ORF11910P	m6A	I
S**C**TCGAKG	41.07%	Yes	M.AhyYL17ORF13945P	m4C or m5C	II
YAAMG^m6^**A**G	99.86%	Yes	AhyYL17I	m6A	II

^a^ Modified bases are highlighted in bold.

### Nucleotide sequence accession numbers.

The complete genome sequence of *Aeromonas hydrophila* YL17 is available in GenBank under the accession number CP007518. The version described in this paper is the second version, CP007518.2.
